# INNOVATIVE APPROACHES TO EVALUATING THE LIFESPAN ASSOCIATIONS OF STRESS, HEALTH, AND WELL-BEING

**DOI:** 10.1093/geroni/igac059.578

**Published:** 2022-12-20

**Authors:** Lewina Lee

## Abstract

Despite widespread agreement on the importance of stress in health and aging, the mechanisms by which psychosocial stressors influence emotional and physical health are not fully understood. Thorough operationalization of stressor exposure and stress response can contribute to a “common language of stress” (Epel et al., 2018) across disciplines, and a lifespan approach can inform the developmental timing of stress-health mechanisms. Guided by these considerations, this symposium presents five studies led by early-career researchers to delineate stress-health associations across the lifespan. Dr. Olivia Atherton will leverage father-offspring data over 25+ years to examine domains of early life stressors that are susceptible to intergenerational transmission and modifiers of such transmission. Drawing from daily diary bursts embedded within a longitudinal study, Dr. Emily Willroth will report on the role of affective reactivity to daily stressors as potential mediators of the association between childhood psychosocial stressors and all-cause mortality risk. Dr. Meaghan Barlow will present on a novel emotion construct – emotion globalizing – by considering the extent to which emotional response to daily stressors influences global assessment of well-being and age differences in these processes. Dr. Soomi Lee will consider job characteristics linked to 10-year stability and change in sleep health profiles in a national adult sample. Dr. Victoria Marino will illustrate a novel approach to assess the flexibility with which individuals select strategies for coping with stressors and describe its association with mortality. Altogether, this symposium contributes evidence on how psychosocial stressors may shape health and well-being across the lifespan.

